# Multiline orthogonal scanning temporal focusing (mosTF) microscopy for scattering reduction in in vivo brain imaging

**DOI:** 10.1038/s41598-024-57208-6

**Published:** 2024-05-13

**Authors:** Yi Xue, Josiah R. Boivin, Dushan N. Wadduwage, Jong Kang Park, Elly Nedivi, Peter T. C. So

**Affiliations:** 1 Department of Mechanical Engineering, Massachusetts Institute of Technology, Cambridge, MA 02139 USA; 2 Laser Biomedical Research Center, Massachusetts Institute of Technology, Cambridge, MA 02139 USA; 3 Picower Institute, Massachusetts Institute of Technology, Cambridge, MA 02139 USA; 4 Department of Biological Engineering, Massachusetts Institute of Technology, Cambridge, MA 02139 USA; 5 Department of Biology, Massachusetts Institute of Technology, Cambridge, MA 02139 USA; 6 Department of Brain and Cognitive Sciences, Massachusetts Institute of Technology, Cambridge, MA 02139 USA; 7 Center for Advanced Imaging, Faculty of Arts and Sciences, Harvard University, Cambridge, MA 02138 USA; 8 Department of Biomedical Engineering, University of California, Davis, CA 95616 USA

**Keywords:** Multiphoton microscopy, Synaptic plasticity, Imaging and sensing

## Abstract

Temporal focusing two-photon microscopy has been utilized for high-resolution imaging of neuronal and synaptic structures across volumes spanning hundreds of microns in vivo. However, a limitation of temporal focusing is the rapid degradation of the signal-to-background ratio and resolution with increasing imaging depth. This degradation is due to scattered emission photons being widely distributed, resulting in a strong background. To overcome this challenge, we have developed multiline orthogonal scanning temporal focusing (mosTF) microscopy. mosTF captures a sequence of images at each scan location of the excitation line. A reconstruction algorithm then reassigns scattered photons back to their correct scan positions. We demonstrate the effectiveness of mosTF by acquiring neuronal images of mice in vivo. Our results show remarkable improvements in in vivo brain imaging with mosTF, while maintaining its speed advantage.

## Introduction

Synaptic remodeling facilitates changes in the connectivity of neural circuits. Imaging this process in vivo is essential for understanding the mechanisms of learning, memory formation, and consolidation. Additionally, it provides insights into the neuropathology of Alzheimer’s disease and other neurodegenerative diseases. Using two-photon laser-scanning microscopy (TPLSM)^1,2^, we have revealed the daily structural dynamics of synapses^3,4^. To monitor their hourly structural dynamics without excessively burdening mice with anesthesia, we aim to develop a rapid two-photon imaging technique. This technique must offer high resolution and a high signal-to-noise ratio, while being suitable for long-term synaptic imaging in vivo. However, imaging synapses on dendritic shafts and spines is more challenging than imaging neuronal somas, due to their smaller and dimmer nature. As a result, synaptic imaging requires higher resolution and a good signal-to-noise level, especially in the presence of substantial multiple scattering. Furthermore, the fluorescence expression level on synapses is lower than on somas, necessitating a higher demand for image signal-to-noise ratio.

To address the challenges of chronic synaptic imaging in vivo, line-scanning temporal focusing microscopy (lineTF) has been developed and utilized for synaptic imaging in the mouse brain^5,6^, as an alternative to TPLSM. lineTF focuses light spatially along one axis and modulates pulse dispersion along the other axis, employing the principle of “temporal focusing”^7–9^. Unlike point-scanning, line scanning in lineTF significantly improves imaging speed by more than an order of magnitude^5^. However, even though lineTF shares the same excitation point spread function (PSF) as TPLSM, its theoretical resolution has not been fully realized in vivo. Due to its reliance on a camera for whole-field image detection, lineTF is heavily impacted by the tissue scattering of emission photons^10^. This scattering causes emission photons to be distributed over a wide range of pixels adjacent to the scan locations, decreasing signal intensity and increasing background noise. Consequently, both the signal-to-noise ratio (SNR) and signal-to-background ratio (SBR) of lineTF are greatly compromised compared to TPLSM.

Many methods have been developed to image or focus light through turbid media^11^. For instance, optical phase-conjugation has been utilized to enhance light transmission through rabbit ears^12^ and mouse skin^13^. This technique gathers transmission photons and operates on the assumption that the scattering process is time-reversible, an approach not feasible for mouse brain imaging. Other methods focus on collecting back-scattered photons for turbidity suppression, yet they require either iterative illumination^14^ or angular scanning^15^ to record tens to hundreds of images for reconstruction. However, the utility of these methods in living tissues is constrained by the millisecond-scale speckle decorrelation times^16^. Furthermore, although these methods^12–15^ have succeeded in focusing light or imaging low-resolution targets through living tissues, they have not yet been applied to image internal structures of tissue at diffraction-limit level resolution.

Structured illumination, combined with image processing, is used to reduce out-of-focus scattering in temporal focusing microscopy^5,17–22^. With just one structured illuminated image^5,17–19^, only ballistic photons are utilized for image reconstruction; the scattered emission photons are simply discarded through low-pass filtering. This approach enhances image contrast but cannot retrieve information carried by the scattered emission photons. In contrast, using multiple structured illuminated images^20–22^, typically ranging from hundreds to thousands, allows the recovery of an object’s image at the resolution of the excitation, independent of image blurring caused by the scattering of the emission photons. While these methods are effective in reducing aberration and scattering, the imaging speed is compromised in order to accurately recover image features.

**Figure 1:**
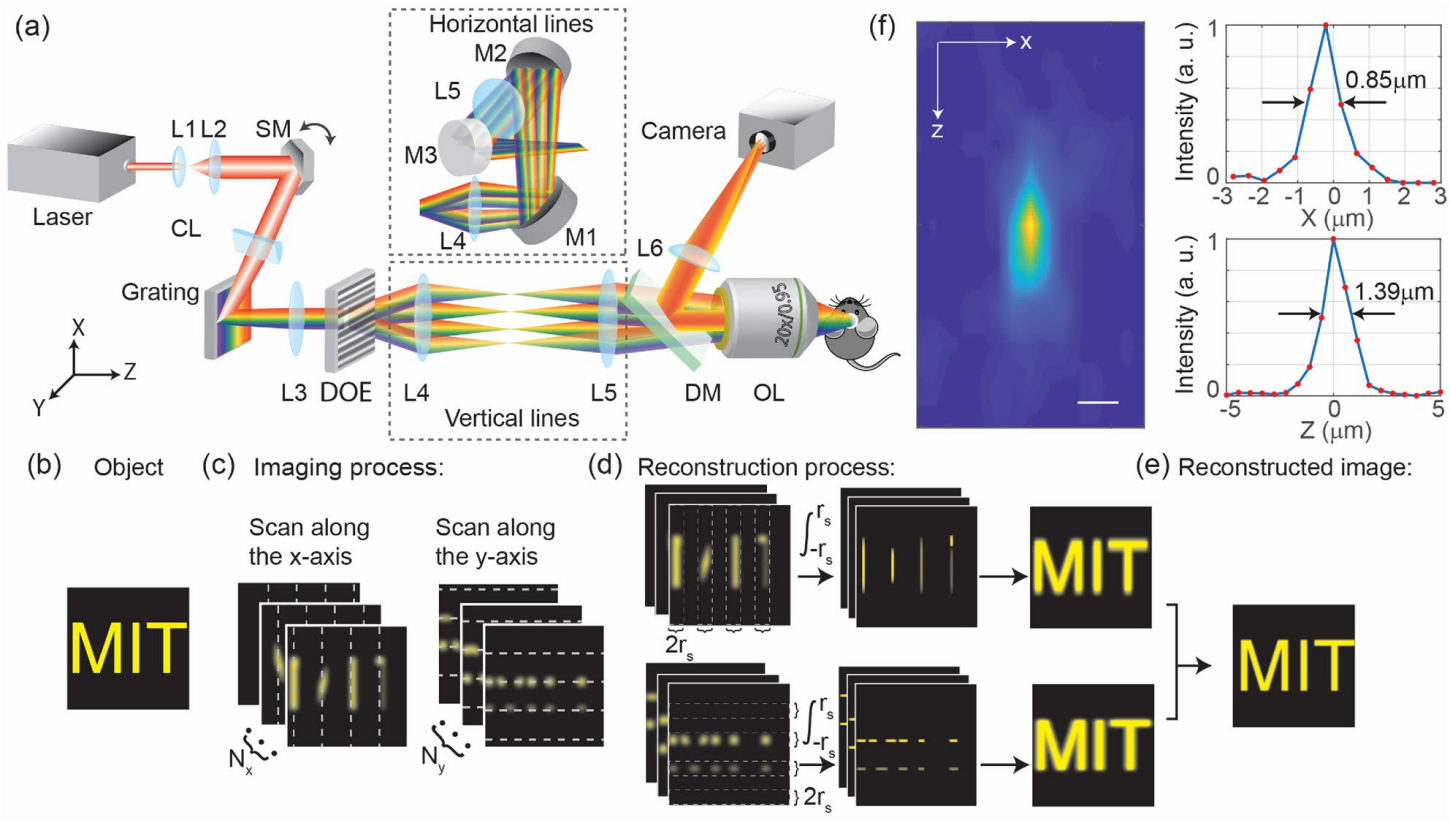
Schematic of mosTF imaging system. (**a**) mosTF setup. Flip mirrors (M1-M3) are utilized to direct light along two alternative paths, thereby rotating the scanning orientation (indicated by the dashed box). Refer to the Methods section for detailed information about the setup. (**b**–**e**) mosTF reconstruction process. (**b**) The object to be imaged through scattering media. (**c**) mosTF captures each intermediate image at every scan position. The dashed lines indicate the locations of the four scanning lines. (**d**) The algorithm then sums scattered photons within the adjacent area (*r*_*s*_) back to the scanning line position. This process is repeated for all *n* intermediate images collected during scanning in both the vertical and horizontal directions. (**e**) The combination of reconstructed images from both directions results in the final image. (**f**) Measured PSF of mosTF (left) and the corresponding lateral and axial intensity profiles of the PSF (right top and bottom, respectively). Fluorescent nanoparticles of 200 nm size were used for this measurement. Scale bar, 1 µm.

Here, we have developed Multiline Orthogonal Scanning Temporal Focusing (mosTF) microscopy to overcome the problem of emission photon scattering in turbid tissue, while largely maintaining the speed advantage of temporal focusing over TPLSM. This technique involves scanning several excitation lines, either horizontally or vertically, to cover the entire field of view (FOV) (see Fig. 1a for the setup and refer to the Methods section for details). Crucially, at each scan location, a fast EMCCD camera captures an intermediate image. Thus, the effect of emission photon scattering can be largely compensated for, similar to TPLSM, by reassigning scattered photons back to their excitation locations. This process enables the reconstruction of a final image with high SBR and resolution (see Fig. 1b–e for the reconstruction process and refer to the Methods section for details). We achieved a spatial resolution of 0.85 µm laterally and 1.39 µm axially (see Fig. 1f). However, unlike point scanning, line scanning introduces additional challenges in photon reassignment and image anisotropy. Reconstructing images from two orthogonally scanned intermediate images mostly restores isotropy in the image (see Fig. S1). After reconstruction, mosTF reveals small structures that would remain invisible with lineTF, thanks to a significantly improved image SBR. We demonstrate mosTF's efficacy by imaging neurons in vivo in mice under anesthesia. mosTF can image dendrites and dendritic spines over a large FOV in the cortical layer 2/3 (approximately at 170 µm depth in the brain), achieving four times the SBR of lineTF and an eightfold faster imaging speed compared to TPLSM. mosTF could be an invaluable tool for real-time visualization of synaptic dynamics, a fundamental aspect of brain development and plasticity^2–4,23,24^.

## Results

### Imaging through scattering media

**Figure 2:**
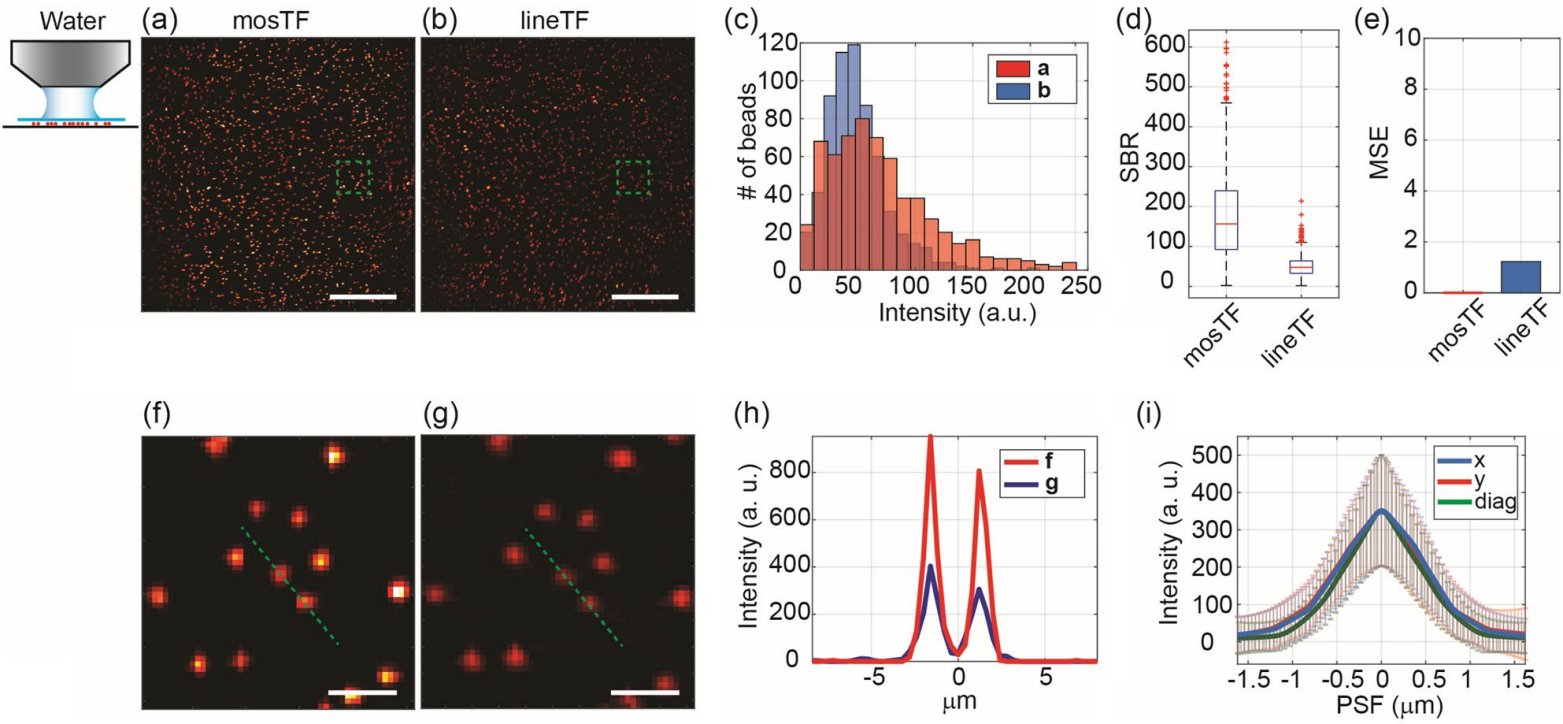
Images of 200 nm beads through water captured by mosTF and lineTF. The objective's working distance is 2 mm. The full FOV is 205 × 205 µm^2^. Images (**a, b, f, g**) are displayed with the same color scale [0–300]. (**a**) mosTF image of the full FOV. (**b**) lineTF image of the full FOV. (**c**) Intensity distribution of beads in (**a**, red) and (**b**, blue). (**d**) SBR comparison (n = 619). The median SBR of mosTF is 156.66, and that of lineTF is 47.62. (**e**) MSE comparison. The mosTF image serves as a reference, resulting in an MSE of zero for mosTF. The MSE of lineTF is 1.23. (**f**, **g**) Zoomed-in views of the area marked by a green box in (**a**) and (**b**), respectively. (**h**) Intensity profile along the cross-section of the two beads in (**f**) and (**g**), indicated by the green dashed line. (**i**) PSF profile along the x- (blue), y- (red), and diagonal (green) directions (n = 295). Full Width at Half Maximum (FWHM) values: FWHMx: 1.15 ± 0.26 µm; FWHMy: 1.15 ± 0.26 µm; FWHMdiag: 1.00 ± 0.24 µm. Scale bar in (**a**, **b**), 50 µm. Scale bar in (**f**, **g**), 5 µm.

**Figure 3:**
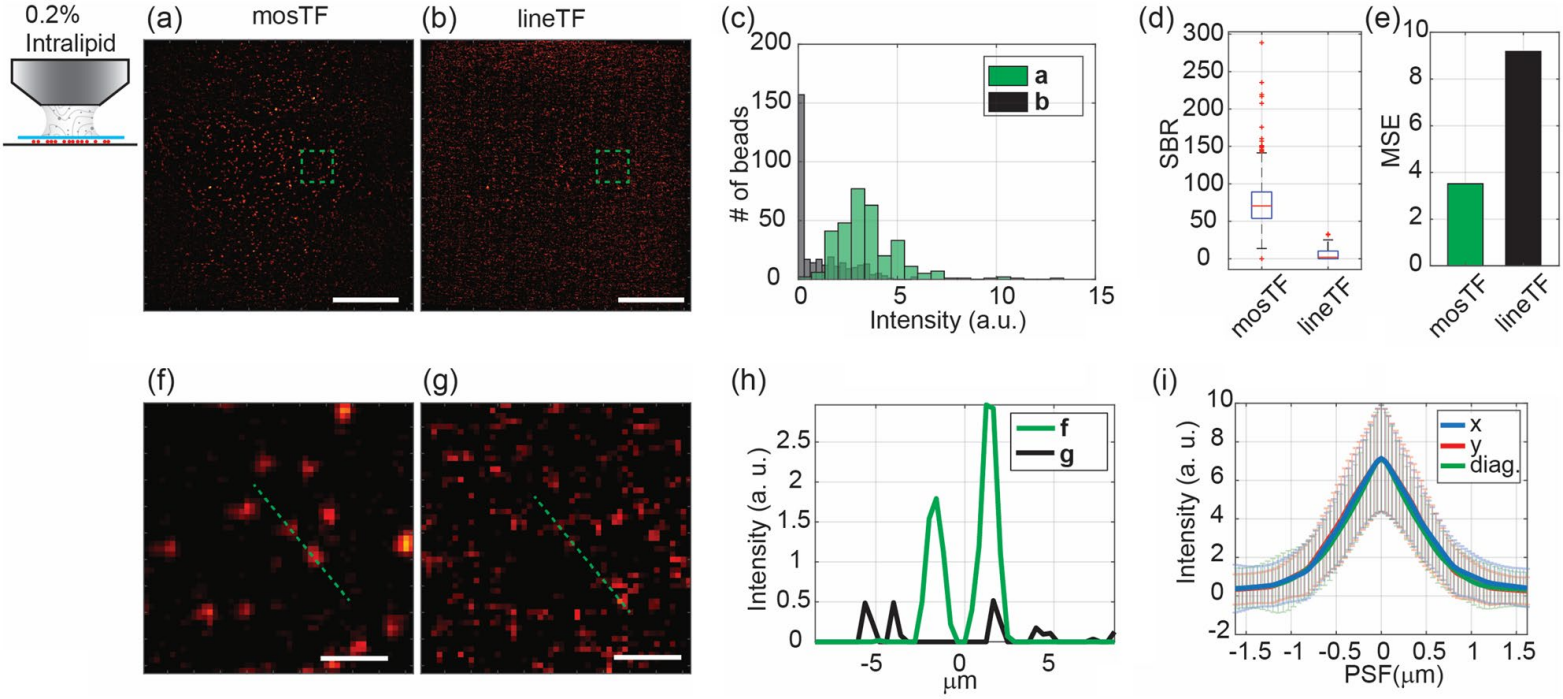
Images of 200 nm beads through a 0.2% intralipid solution captured by mosTF and lineTF. mosTF effectively reduces the scattering caused by the turbid medium. The objective’s working distance is 2 mm, therefore, the thickness of the scattering solution is 2 mm. The full FOV is 205 × 205 µm^2^. Images (**a**, **b**, **f**, **g**) are displayed with the same color scale [0–9]. (**a**) mosTF image of the full FOV. (**b**) lineTF image of the full FOV, where the beads are barely distinguishable. (**c**) Intensity distribution of beads in (**a**, green) and (**b**, black). (**d**) SBR comparison (n = 320). The median SBR of mosTF is 70.448, and that of lineTF is 1.954. (**e**) MSE comparison with respect to the mosTF image with water immersion. MSE of mosTF: 3.53; MSE of lineTF: 9.18. (**f**, **g**) Zoomed-in views of the area marked by a green box in (**a**) and (**b**), respectively. (**h**) Intensity profile along the cross-section of the two beads in (**f**) and (**g**), indicated by the green dashed line. (**i**) PSF profile along the x- (blue), y- (red), and diagonal (green) directions (n = 346). FWHM values: FWHMx: 1.01 ± 0.35 µm; FWHMy: 1.00 ± 0.30 µm; FWHMdiag: 0.91 ± 0.29 µm. Scale bar in (**a**, **b**), 50 µm. Scale bar in (**f**, **g**), 5 µm.

As a first experiment, we imaged the same area of fluorescent beads using water (which causes minimal scattering, as shown in Fig. 2) and then a 0.2% intralipid solution (which mimics tissue-level dynamic scattering, as shown in Fig. 3) as the immersion medium for the objective. After acquiring raw intermediate images using parallel scanning with four lines, the mosTF images were reconstructed by photon reassignment using the algorithm detailed in the Methods section. The images without reconstruction, referred to as the lineTF images, were created by directly summing the intermediate images without applying photon reassignment.

For the control experiment using water as the immersion medium, we first quantified image quality by evaluating the intensity distribution of the beads, SBR, and Mean-Square-Error (MSE) across the large FOV (Fig. 2a,b). The SBR, also referred to as image contrast, is calculated by dividing the average intensity of the signals by the average intensity of the background. MSE calculates the average squared difference between the reconstructed image and the ground truth image to assess the effectiveness of the reconstruction. The intensity distributions of the beads were nearly identical for both mosTF and lineTF images (Fig. 2c). The median SBR for mosTF improved threefold after reconstruction (Fig. 2d). Even with a minimal scattering medium, photons from a point source were still detected by multiple pixels. The reconstruction process resembled binning. MSE is calculated with respect to the mosTF image. The MSE for the lineTF image is 1.23, attributable to noise in the image (Fig.2e). A zoomed-in view of individual beads (Fig. 2f–h) shows an improvement in signal intensity. The reconstructed PSF is isotropic (Fig. 2i) due to orthogonal scanning and combination. The intermediate images with horizontal and vertical scanning, as well as their corresponding Fourier domain images, are shown in Supplementary Fig. 1. In summary, mosTF slightly improves image SBR compared to lineTF when water is used as a minimal scattering immersion medium.

We subsequently compared bead images captured by mosTF and lineTF using a 0.2% intralipid solution to simulate the effects of dynamic tissue scattering (Fig. 3). Statistical analysis of the full FOV clearly demonstrates mosTF’s advantage (Fig. 3a–e). When imaging through a 2 mm 0.2% intralipid solution (approximately $3.2l_{s}^{em}$ where $l_{s}^{em}$ is the mean free path length of the emission photons; see calculations in Methods), lineTF barely detects signals from the beads, while mosTF successfully identifies individual beads (Fig. 3c). Compared to lineTF, mosTF improves the SBR by 36 times (Fig. 3d). The median SBR of mosTF images through turbid media is 70.45, which is even higher than that of lineTF images in the absence of turbid media (47.61). Considering the mosTF image with water immersion, the MSE of mosTF with lipid immersion is 3.53, and for lineTF with lipid immersion, it is 9.18 (Fig. 3e). These MSE results suggest that mosTF images capture similar structural information to those taken through water immersion, while lineTF images lose substantial structural information. Beyond the analysis of the full FOV, we also examined individual beads in a zoomed-in view (Fig. 3f–i). The intensity profile reveals that lineTF cannot differentiate between signal and noise in this scenario, whereas mosTF still distinctly resolves two adjacent beads (Fig. 3h). The PSF measurements along three different directions (x, y, and diagonal) are nearly identical (Fig. 3i), confirming that the reconstructed PSF is isotropic. Therefore, mosTF significantly enhances SBR and retains most of the structural information when imaging through turbid media.

### In vivo brain imaging of mice under anesthesia

We tested the performance of mosTF microscopy for in vivo imaging in the brain of an anesthetized mouse through a cranial window (refer to the animal procedure in the Methods section). The sample is highly heterogeneous, comprising layers of different materials: cover glass, meninges, brain matter, and blood vessels. Additionally, biological processes such as muscle contractions from breathing and heartbeat can introduce unpredictable tissue movement^12,13^. Even though it is challenging, since mosTF is capable of imaging through turbid media without prior measurement, it can also image through dynamic scattering tissue, provided that the dynamics are slower than the time required to complete the two orthogonal scans. Furthermore, the mosTF reconstruction process treats each diffraction-limited region individually, making it robust against spatially varied turbid media.

**Figure 4:**
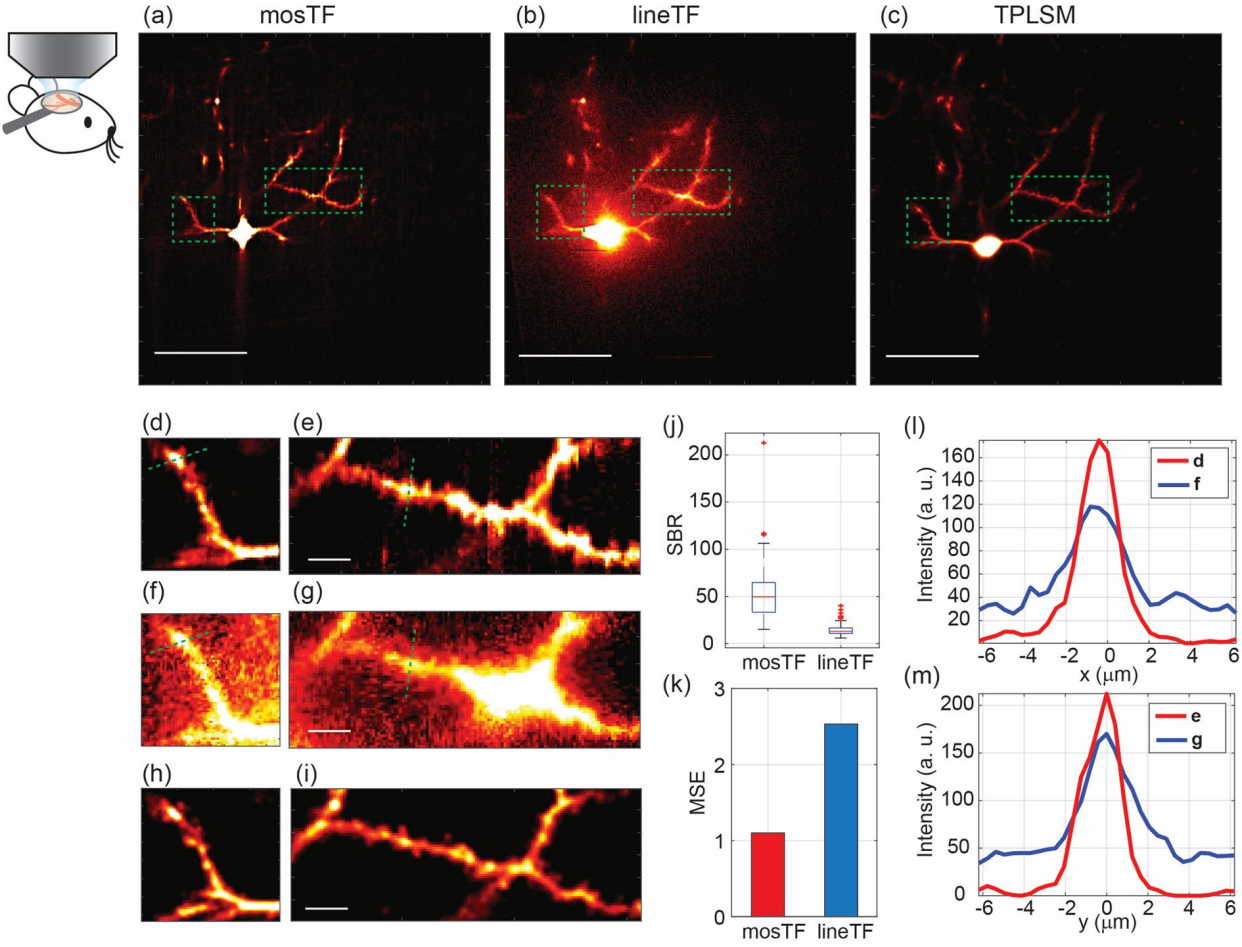
mosTF effectively overcomes spatial and time-dependent scattering for in vivo brain imaging. The imaged neuron is located approximately 170 µm deep from the surface of the mouse brain (pia mater). (**a**–**c**) Full FOV images of the same cell captured by mosTF, lineTF, and TPLSM. Scale bar, 50 µm. Images are displayed with the same color scale [0–250]. (**d**–**i**) Zoomed-in views of (**a**–**c**). The mosTF image (**d**,**e**) clearly shows spines on the dendritic shaft. Images are displayed with the same color scale [0–100]. Scale bar, 5 µm. (**j**,**k**) Statistical analysis of the performance of mosTF and lineTF. (**j**) SBR comparison. The median SBR for mosTF is 49.557, while for lineTF it is 13.082. (**k**) MSE comparison between mosTF and lineTF. The MSE for the mosTF image is 1.10, and for lineTF, it is 2.53. The TPLSM image is used as the reference for MSE calculation. (**l**–**m**) Intensity profiles of (**l**) the approximate horizontal cross-section and (**m**) the approximate vertical cross-section, marked by the green dashed line in (**d**, **f**) and (**e**, **g**). Blue line represents lineTF; red line represents mosTF.

We imaged layer 2/3 pyramidal neurons labeled with eYFP as cell fill in an anesthetized mouse using mosTF, lineTF, and then TPLSM to generate a “ground truth” image. A focal plane containing both soma and dendritic spines, which carry excitatory synapses and are located at approximately 170 µm depth inside the mouse brain (about $3l_{s}^{em}$, with calculations provided in the Methods section), is shown as a demonstration. The pixel size is 0.4 µm, and the FOV is approximately 205 × 205 µm^2^ (Fig. 4a,b). The TPLSM image (Fig. 4c) shares the same FOV, with a pixel size of 0.25 µm. The signal from dendritic spines is about 10–20 photons, while the soma emits about 500 photons (as measured from the TPLSM image). In the lineTF image, scattered photons from the soma are brighter than the nearby spines, making the signal from the spines indistinguishable from background noise (Fig. 4f,g). In contrast, mosTF reassigns the scattered photons back to their origin, clearly revealing the spines on the dendritic shaft (Fig. 4d,e). To quantitatively demonstrate mosTF’s advantage, the SBR was measured from 49 individual locations, including soma, dendrites, and spines. The median SBR of mosTF (49.56) is nearly four times higher than that of lineTF (13.08) (Fig. 4j). To evaluate the structural similarity of fine structures, we also calculated the MSE of mosTF and lineTF images with respect to the TPLSM image (Fig. 4k) in the region of dendrites (Fig. 4e,g,i). The mosTF image has a better MSE (1.10) compared to the lineTF image (2.53), indicating that mosTF recovers most fine structures, such as dendrites and spines, while also reducing background noise compared to lineTF. The SBR and MSE results demonstrate that mosTF effectively reassigns scattering photons to the correct pixels. Moreover, orthogonal scanning reduces scattering and uniformly extends frequency coverage in both horizontal (green dashed line, Fig. 4d,f) and vertical directions (green dashed line, Fig. 4e,g), as shown in the representative intensity profiles in both the x and y directions (Fig. 4l–m). Most importantly, the intensity profiles of mosTF and lineTF reveal that mosTF not only increases signal intensity but also reduces background noise. Consequently, mosTF’s effective photon reassignment leads to an improvement in both SBR and the fidelity of structural information. Therefore, mosTF is capable of resolving small structures, such as dendritic spines, within a large FOV in vivo.

## Discussion

By reassigning scattered photons, mosTF significantly improves the SBR and preserves fine structures when imaging through turbid media, outperforming lineTF. mosTF is capable of imaging eight times faster than TPLSM while maintaining the same spatial resolution and high SBR (see calculations in the Methods section). We have demonstrated mosTF’s effectiveness by imaging 200 nm red fluorescent beads through a 2 mm thick 0.2% intralipid solution. While the lineTF image fails to distinguish any beads, the mosTF image clearly distinguishes individual beads with high SBR. We also present in vivo brain imaging in an anesthetized mouse, conducted without any prior correction for scattering. mosTF can distinctly identify dendritic spines, which are approximately 1 μm in size, at a depth of around 170 μm and across a 205 × 205 μm^2^ FOV in the mouse brain. In contrast, these structures are mostly obscured by scattered photons from adjacent bright areas in lineTF images. The purpose of this experiment is to demonstrate the improvement in the SNR with mosTF rather than to explore the maximum accessible depth, because the maximum accessible depth depends on both optical techniques and the fluorescence labeling of the samples. The effect of scattering reduction could be further enhanced with a camera that has lower read noise and dark current. In conclusion, mosTF offers advantages in terms of SBR and MSE over lineTF for in vivo imaging, and also achieves isotropic scattering reduction through orthogonal scanning.

Compared to other post-processing methods that aim to reduce scattering in temporal focusing^5,17–22^, mosTF uniquely reassigns scattered photons back to their original positions instead of merely discarding them. Previous works^5,17–22^ utilizing structured or patterned illumination techniques take multiple images under varying illumination conditions. In these methods, only in-focus information encoded by the pattern is preserved, while out-of-focus scattered photons are eliminated by the post-processing algorithm. These techniques increase the SBR but not the SNR, as they reduce the intensity of the background without increasing the signal intensity. Here, SNR is calculated by dividing the average intensity of signals by the average intensity of noise, predominantly Poisson noise in fluorescence images. However, mosTF not only decreases background noise caused by scattered photons but also increases signal intensity by reassigning these photons. This ability to reassign photons is the reason for the substantial improvement in both SBR and SNR observed with mosTF. It enables the visualization of small, weakly fluorescent structures that remain invisible with lineTF.

The imaging speed of mosTF could potentially be further enhanced in two ways: by using a camera with a faster imaging speed and a laser with a shorter pulse duration. As discussed in the Methods section, with a faster and lower readout noise camera, the theoretical speed improvement of mosTF, utilizing our four-line scanning strategy, could be approximately 40-fold compared to TPLSM. Currently, we have achieved an eight-fold speed improvement, which is limited by the camera we used. If imaging brighter fluorescence samples, such as somas instead of spines, the imaging speed could be increased even further to achieve sufficient SNR (refer to the calculations in the Methods section for details). The second factor in improving mosTF imaging speed is the number of parallel lines. We can reduce the pulse duration of the laser from 200 to 50 fs by implementing dispersion compensation. With a pulse duration four times shorter, only half of the power per line is needed to achieve the same level of two-photon excitation efficiency^25^. This implies that the number of parallel lines could be doubled while using the same total power, which is constrained by the tissue's thermal damage threshold (typically less than 200 mW). These two factors could, in total, potentially improve the imaging speed of mosTF by a factor of 10 with upgrades to both the camera and the laser.

According to the theory (refer to the reconstruction algorithms in the Methods section), one-dimensional reconstruction results in an anisotropic PSF for mosTF. As a proof-of-principle setup, we have implemented orthogonal scanning that rotates the beam by *π*/2 using mirrors. The current PSF exhibits a slight “cross” shape, but it could be made more isotropic with additional rotation angles. Beam rotation at arbitrary angles can be achieved using a Dove prism, coupled with pre-chirp modulation, to maintain a short pulse duration.

In summary, mosTF represents a novel approach for fast two-photon imaging through scattering media. It offers improved signal-to-background ratio and maintains structural information more effectively compared to line-scan temporal focusing microscopy, while also achieving a speed advantage over point-scan two-photon microscopy. mosTF is well-suited for in vivo imaging of dynamic events in small structures across a large field of view within scattering tissues, such as the brain. This potentially facilitates studies in brain plasticity.

## Methods

### Ethical statement

All animal procedures were approved by the Institutional Animal Care and Use Committees at the Massachusetts Institute of Technology and meet the National Institutes of Health (NIH) guidelines for the care and use of vertebrate animals.

### mosTF reconstruction algorithm

lineTF in the presence of emission scattering can be readily modeled. mosTF applies several excitation lines scanned in parallel at either horizontal or vertical orientations combined via the reconstruction algorithm. Here, we focus on a 2D reconstruction. We assume that the excitation PSF (*P*_*ex*_) is not affected by the specimen. This assumption is mostly valid within 200 µm depth inside the mouse brain, because the tissue scattering and aberration of infrared excitation light is minimal^26^. We treat the emission PSF (*P*_*em*_) as unknown and potentially spatially varied due to local tissue heterogeneity. Therefore, the general form of an intermediate image *I*_1_ (Fig. 1c,d, first column) at a scanning location *x*_*i*_ for a vertical line scanned along the *x*-axis within the FOV (*N*_*x*_ × *N*_*y*_, $0 \le x_{i} \le N_{x}$) can be written as:

1 $$I_{1} \left( {x,y; x_{i} } \right) = O\left( {x,y} \right)[E(x,y; x_{i} ) \otimes P_{ex} \left( {x,y} \right)] \otimes P_{em} \left( {x,y;x_{i} } \right),$$

where *P*_*ex*_(*x*, *y*) is the excitation PSF, $P_{em} \left( {x,y;x_{i} } \right)$ is the emission PSF that can vary dependent on excitation location, *O*(*x*, *y*) is the fluorophore distribution, $E\left( {x,y;x_{i} } \right)$ is the excitation line pattern, and ⊗ denotes convolution.

Since scanning along the *x* or *y*-axis is entirely equivalent, we will only provide details for the scan along the *x*-axis, until the final steps where the two sets of images are combined.

We first consider a single scanning line at position *x*_*i*_. Thus, $E\left( {x,y;x_{i} } \right) = \delta \left( {x - x_{i} } \right)$. The intermediate image *I*_1_ in Eq. (1) can be written as:

2 $$\begin{aligned} I_{1} \left( {x,y;x_{i} } \right) & = O\left( {x,y} \right)\left[ {\delta \left( {x - x_{i} } \right) \otimes P_{ex} \left( {x,y} \right)} \right] \otimes P_{em} \left( {x,y;x_{i} } \right) \\ & = O\left( {x,y} \right)P_{ex} \left( {x - x_{i} ,y} \right) \otimes P_{em} \left( {x,y;x_{i} } \right) \\ & = \iint {O\left( {x^{\prime } ,y^{\prime } } \right)P_{ex} \left( {x^{\prime } - x_{i} ,y^{\prime } } \right)P_{em} \left( {x - x^{\prime } ,y - y^{\prime } ;x_{i} } \right)dx^{\prime } dy^{\prime } } \\ \end{aligned}$$

Because only fluorophores along the excitation line $P_{ex} \left( {x - x_{i} ,y} \right)$ are excited and not anywhere else, in the corresponding intermediate image $I_{1} \left( {x,y;x_{i} } \right)$, all the fluorescence detected by different pixels on the camera must originate from the excitation line $P_{ex} \left( {x - x_{i} ,y} \right)$ in the specimen at location *x*_*i*_. Therefore, we can reassign the scattered emission photons by summing all the photons detected outside of the excitation line at location *x*_*i*_.

Theoretically, emission fluorescence photons should be integrated across the whole FOV. However, integral of pixels where photon counts are below readout noise of the camera only adds readout noise rather than signal to the reconstructed image. To optimize the SNR, only pixels with intensity above readout noise were integrated. Within the same FOV, scattered photons from a brighter object will still be detectable even far away from the original excitation location compared to a dimmer object, resulting in a larger effective region. Therefore, we applied an adaptive interval of the integral $r_{s} \left( {x_{i} ,y} \right)$, which varies spatially depending on the local intensity. We can model this integral process as

3 $$\begin{aligned} I_{2} \left( {y;x_{i} } \right) & = \mathop \int \limits_{{x_{i} - r_{s} \left( {x_{i} , y} \right)}}^{{x_{i} + r_{s} \left( {x_{i} , y} \right)}} I_{1} \left( {x,y;x_{i} } \right)dx \\ & = \iint {O\left( {x^{\prime } ,y^{\prime } } \right)P_{ex} \left( {x^{\prime } - x_{i} ,y^{\prime } } \right) \left[ {\mathop \int \limits_{{x_{i} - r_{s} \left( {x_{i} , y} \right)}}^{{x_{i} + r_{s} \left( {x_{i} , y} \right)}} P_{em} \left( {x - x^{\prime } ,y - y^{\prime } ;x_{i} } \right)dx} \right]dx^{\prime } dy^{\prime } } \\ & = \iint {O\left( {x^{\prime } ,y^{\prime } } \right)P_{ex} \left( {x^{\prime } - x_{i} ,y^{\prime } } \right)\tilde{P}_{em} \left( {x^{\prime } ,y - y^{\prime } ;x_{i} } \right)dx^{\prime } dy^{\prime } }, \\ \end{aligned}$$

where

4 $$\tilde{P}_{em} \left( {x^{\prime } ,y - y^{\prime } ;x_{i} } \right) = \mathop \int \limits_{{x_{i} - r_{s} \left( {x_{i} , y} \right)}}^{{x_{i} + r_{s} \left( {x_{i} , y} \right)}} P_{em} \left( {x - x^{\prime } ,y - y^{\prime } ;x_{i} } \right)dx$$

is the reconstructed emission PSF after photon reassignment by 1D integral.

In Eq. (3), $I_{2} \left( {y;x_{i} } \right)$ is a single reconstructed line at the scanning location *x*_*i*_ (Fig. 1d, middle column). As we scan across the entire FOV, we can apply the operation described in Eqs. (1–4) to all intermediate image $I_{1} \left( {x, y;x_{i} } \right)$ and achieve reconstructed lines $I_{2} \left( {y;x_{i} } \right)$, where $0 \le x_{i} \le N_{x}$. Next, we generate a composite image $I_{3}^{x} \left( {x, y} \right)$, which is an augmented matrix created by appending all reconstructed lines $I_{2} \left( {y;x_{i} } \right)$, where $0 \le x_{i} \le N_{x}$, written as

5 $$I_{3}^{x} \left( {x, y} \right) = I_{2} \left( {y;x} \right).$$

For $I_{3}^{x} \left( {x, y} \right)$, scattered fluorescence photons are reassigned along the *x*-axis but not along the *y*-axis after scanning along the *x*-axis across the entire FOV. Therefore, $I_{3}^{x} \left( {x, y} \right)$ is the reconstructed image along only the *x*-axis (Fig. 1d, last column). Similarly, we can generate a composite image reconstructed along only the *y*-axis:

6 $$I_{3}^{y} \left( {x, y} \right) = I_{2} \left( {x;y} \right).$$

Because the reassignment is effectively only along one dimension (as shown in Fig. S1a,b), their Fourier space coverages are anisotropic (illustrated in Fig. S1d,e). Specifically, since $I_{3}^{x} \left( {x, y} \right)$ reassigns fluorescent photons along the x-axis (Fig. S1b), it recovers higher spatial frequencies along the *k*_*x*_-axis in the Fourier domain (Fig. S1e). Similarly, $I_{3}^{y} \left( {x, y} \right)$ achieves higher resolution along the y-axis (Fig. S1a), and correspondingly, recovers higher spatial frequencies along the *k*_*y*_-axis (Fig. S1d). An image $I_{4} \left( {x, y} \right)$ with improved and isotropic resolution can be reconstructed by combining $I_{3}^{x} \left( {x, y} \right)$ and $I_{3}^{y} \left( {x, y} \right)$ in the Fourier domain (Fig. S1c). Specifically,

7 $$I_{4} \left( {x, y} \right) = {\Im }^{ - 1} \left( {w{\Im }\left( {I_{3}^{x} \left( {x, y} \right)} \right) + \left( {1 - w} \right){\Im }(I_{3}^{y} \left( {x, y} \right)} \right)),$$

where ${\Im }$ and ${\Im }^{ - 1}$ denote 2D Fourier transform and its inverse. A weighting factor *w*, typically around 0.5, is added to balance the frequency coverage in the two directions. This factor accounts for intensity differences between the two sets of images that may arise due to effects such as misalignment and photobleaching. For example, the Fourier transform of $I_{3}^{y} \left( {x, y} \right)$ (Fig. S1d) appears dimmer than that of $I_{3}^{x} \left( {x, y} \right)$ (Fig. S1e) due to misalignment. As shown in Fig. S1f, the Fourier transform of $I_{4} \left( {x, y} \right)$ exhibits a more circularly symmetric coverage in the Fourier domain, resulting in improved and isotropic resolution in the reconstructed image $I_{4} \left( {x, y} \right)$. In addition, median filters were applied to remove random noise, and edge enhancement filters were used to sharpen fine structures in the Fourier transforms of $I_{3}^{x} \left( {x, y} \right)$ and $I_{3}^{y} \left( {x, y} \right)$ before recombination, which is omitted in Eq. 7 for clarity. The overlay of reconstructed PSF from two orthogonal directions significantly extends the bandwidth coverage along the *k*_*x*_ and *k*_*y*_ axes, going beyond the limits imposed by the emission PSF without scattering correction.

### mosTF setup and imaging parameters

The mosTF system is based on a standard lineTF design (Fig. 1a). The laser generates femtosecond pulses at a wavelength of 1035 nm (repetition rate 1 MHz, pulse width  < 350 fs, maximum pulse energy 40 µJ at 1 MHz, spectral width 10 nm, Monaco, Coherent Inc., CA, USA). The scanning mirror (6350, Cambridge Technology, MA, USA) mechanically scans the beam along the x-axis. The cylindrical lens (*f* = 150 mm) focuses the beam into a line on the grating (20RG1200-1000-2, Newport Co., CA, USA, 1200 grooves/mm, grating efficiency 80% at 1035 nm). The incident angle θ_i_ is about 73°, so the 1st order diffraction angle is about 17°. The grating generates dispersion along the y-axis. L3 (*f* = 300 mm) and L4 (*f* = 75 mm) are relay lenses. A diffractive optical element (MS-635-J-Y-S, HOLO/OR Ltd., Israel) is placed on the conjugate Fourier plane after L3 to generate four parallel lines. The number of parallel lines is calculated from the thermal damage threshold of in vivo imaging and the power required for efficient two-photon excitation of a single diffractive spot. After L4, flip mirrors (M1-M3) are placed before L5 to rotate the scanning direction by 90° for the other scanning direction. L5 (*f* = 300 mm) is the tube lens. On the back focal plane, the beam size is about 20 × 20 mm. We overfilled the back aperture so that the excitation PSF of mosTF microscopy is comparable with TPLSM. The FOV is 205 × 205 µm^2^. L6 (*f* = 350 mm) is the tube lens in the detection path. The system magnification is about 40× according to the objective magnification and the focal length of the tube lenses. The image is detected by an EMCCD camera (HNu 512, Nuvu Cameras, Canada). Lenses are purchased from Thorlabs.

### Image collection for mosTF and lineTF

In the experiments, the EMCCD has 512 × 512 pixels corresponding to a 205 × 205 µm^2^ FOV. To avoid cross-talk between lines but maximize the number of parallel lines used, we chose the number of parallel lines according to the scattered emission PSF. The scattered emission PSF depends on both the mean-free-path of mouse brain^26^ and imaging depth. For our in vivo experiment, we parallelized four lines with 51 µm separation. Therefore, each line needs to scan a region of 128 × 512 pixels. The PSF of the system is 0.85 µm in lateral direction and 1.39 µm in axial direction (Fig. 2f). To fulfill the Nyquist requirement, the pixel size is 0.4 µm. An intermediate image is taken at the position of every pixel. So, the total number of intermediate images is 128 per plane. Based on our previous experiments of single line-scan temporal focusing in vivo^5^, the exposure time required to collect sufficient emission from one imaging plane is 1.6 s. Thus, for four-line scanning, the exposure time required to collect sufficient emission from one plane is 400 ms. During the 400 ms, we need to capture 128 intermediate images. The desired exposure time of each intermediate image is therefore 3 ms. However, the maximum imaging speed of the EMCCD in our experiment is 63 fps (16 ms), which is the current limitation on mosTF imaging speed. Thus, the exposure time of each intermediate image was set to be 16 ms, and the total exposure time per 2D plane was 2 s (16 ms × 128). Afterwards, scanning was repeated along the orthogonal direction, doubling the total imaging time for a total exposure time of 4 s per 2D plane (16 ms × 128 × 2). When compared to the speed of TPLSM^3–5^, which takes about 30 s to collect sufficient emissions from one plane in our in vivo preparation, mosTF shows an eight-fold improvement in imaging speed. Noticed that time comparison between different systems are based on collecting similar number of emission photons. Without the speed limitation imposed by the maximum frame rate of the camera, the theoretical speed improvement of mosTF using our 4-line scanning strategy would be approximately 40-fold compared to TPLSM.

mosTF requires a high frame rate camera with low readout noise. As shown in the “mosTF reconstruction algorithm”, the final image is reconstructed by summing the scattered emission photons on adjacent pixels back to the focal point. The readout noise of the camera should be low enough so that summing all these pixels still maintains single photon sensitivity in the final image. At the same time, the overall imaging time of a single plane depends on the maximum frame rate when the sample is bright enough. The EMCCD (HNu 512, Nuvu Cameras, Canada) used in mosTF has 0.05e^−^ readout noise per frame at the maximum gain. The camera was tested by imaging a blank cover glass to measure the background intensity. We mimicked the lineTF image by directly summing 128 intermediate images without reconstruction. In this case, the background is 2.57e^−^ (± 2.14e^−^), which is similar to a standard lineTF image. After mosTF reconstruction, the background is 0.13e^−^ (± 0.39e^−^) (Fig. S2). If imaging brighter fluorescence samples, such as soma of neuron instead of spines, the exposure time per pixel could be much shorter to achieve sufficient SNR. Recent development of high frame rate intensified sCMOS camera can significantly alleviate camera speed limitation. An alternative approach is to image in a de-scanned geometry. In this approach, each scan line and the associated scanned region are projected to a column at the camera using elliptical optics. Instead of reading out the whole camera, approximately a column for each scan line needs to be readout. In the case of four scan lines, the effective frame rate using the intensified sCMOS camera can exceed 4 kHz. For 512 × 512 image, final maximum frame rate can reach 31 Hz while providing excellent SNR image with over 250 µs pixel dwell time.

### Image collection for TPLSM

TPLSM was performed using a custom-built microscope with a Mai Tai HP Ti: Sapphire laser (Spectra Physics) tuned to 1030 nm as the source of excitation. The power delivered to the specimen ranged from 30 to 50 mW depending on imaging depth. Galvanometric XY scanning mirrors (6215H, Cambridge Technology) and a piezo actuator Z positioning system (Piezosystem Jena) were used for XY and Z movement, respectively. The pixel size was 250 nm in XY, and the Z step size was 1 µm. The dwell time per pixel was 40 µs. The dwell time was determined based on our previous data as the time required to collect sufficient emission photons from the dendrites of L2/3 pyramidal neurons expressing an eYFP cell fill in vivo^3,4^. The beam was focused by a 20x/1.00 NA water immersion objective lens (W Plan-Apochromat, Zeiss). Emissions were collected by the same objective lens, passed through an IR blocking filter (E700SP, Chroma Technology), and separated by dichroic mirrors at 520 nm and 560 nm. After passing through three independent band-pass filters (485/70 nm, 550/100 nm, and 605/75 nm), emissions were collected simultaneously onto three separate PMTs. Raw 2-photon scanning data were processed for spectral linear unmixing and converted into a tif Z stack using Matlab (Mathworks) and ImageJ (NIH). Only the fluorescence assigned to the 550/100 nm (i.e. yellow) channel was used for this study to visualize the dendritic morphology of L2/3 pyramidal neurons expressing an eYFP cell fill.

### Bead sample preparation for scattering and non-scattering imaging conditions

In addition to in vivo specimens, bead samples with known scattering mean free paths were prepared to evaluate the performance of this imaging system. The mean free path length ($l_{s}^{em}$) of emission photons was calculated by $l_{s}^{em} = 1/\mu_{s}$, where *μ*_*s*_ is the scattering coefficient. For in vivo imaging, the brain tissue and blood are a highly heterogeneous. The scattering coefficient of mouse or rat brain varies slightly in the literature^26–28^. The mean free path length $l_{s}^{em}$ at 532 nm is about 43.5–58.8 µm calculated from the reported scattering parameters measured in vitro^26–28^. The anisotropy of mouse brain tissue is about 0.98 according to previous work^29^. These values inform our choice of intralipid concentration and thickness to mimic brain scattering. For a 2% intralipid solution, *μ*_*s*_ equals 16 mm^−1^ according to literature^30^. Since the scattering coefficient is linearly proportional to concentration, $l_{s}^{em}$ of 0.2% intralipid solution is 0.625 mm. Our samples consisted of 200 nm diameter red fluorescent beads (the same as used for the PSF measurement) mounted in clear medium (Fluoromount-G®, SouthernBiotech, AL, USA). The specimens were imaged through 2 mm of water as controls. The same specimen was then imaged through 2 mm of 0.2% intralipid solution as scattering phantoms. These phantoms have scattering equivalent to $3.2l_{s}^{em}$ in the brain or corresponding to a tissue depth of roughly 160 µm.

### In vivo specimen preparation

The study is reported in accordance with ARRIVE guidelines. In vivo experiments with C57NL/6 J mice are to demonstrate the mosTF imaging technique. One mouse was used to generate the in vivo images in the manuscript. Data were not excluded from this manuscript, i.e., one mouse was imaged for demonstration purposes, and those images are shown in the manuscript. Randomization was not relevant to this study, as there were no experimental groups. Blinding was not relevant to this study, as there were no experimental groups. Image signal-to-background ratio (SBR), resolution, and similarity to the ground-truth image are used as outcome measures to assess the effectiveness of mosTF (Fig. 4). Statistical analysis across different animals was not relevant to this study. Statistical analysis of image SBR was calculated from 49 independent locations in the same mouse. One C57BL/6 J mouse, male, age postnatal day 63, was imaged in the experiment.

Experimental procedures: In utero electroporation was performed on embryonic day 15.5 timed pregnant C57BL/6 J mice to label a sparse population of L2/3 pyramidal neurons with an eYFP cell fill, enabling visualization of the neurons’ dendritic morphology. Constructs used for in utero electroporation were a cre-dependent eYFP cell fill (*pFUdioeYFPW*^3^) and a *Cre* plasmid^31^ at concentrations of 0.7 µg/µl and 0.03 µg/µl, respectively, with 0.1% Fast Green for visualization. A total of 0.5–1.0 µl of the plasmid solution was injected into the right lateral ventricle, and five pulses of 36 V (duration 50 ms, frequency 1 Hz) targeting the visual cortex were delivered from a square-wave electroporator (ECM830, Harvard Apparatus). Pups were then reared to adolescence (P44) and implanted with a 5 mm cranial window over the right hemisphere as described previously^32^. After 2 weeks of surgery recovery, animals were fitted with a custom head mount to enable fixation to the microscope stage. All imaging took place under isoflurane anesthesia (1.25%) with the head mount fixed to the microscope stage.

Results: The in vivo experiment demonstrates mosTF is able to resolve small structures, such as dendritic spines, within the large FOV. Compared to lineTF, mosTF not only increases the signal intensity but also reduces the background noise.

### Supplementary Information

Supplementary Figures.

## Data Availability

The data that support the findings of this study are available from the corresponding author, P. T. C. S., upon reasonable request.

## References

[1] Yuste R, Denk W (1995). Dendritic spines as basic functional units of neuronal integration. Nature.

[2] Chen JL, Lin WC, Cha JW, So PT, Kubota Y, Nedivi E (2011). Structural basis for the role of inhibition in facilitating adult brain plasticity. Nat. Neurosci..

[3] Chen JL, Villa KL, Cha JW, So PT, Kubota Y, Nedivi E (2012). Clustered dynamics of inhibitory synapses and dendritic spines in the adult neocortex. Neuron.

[4] Villa KL, Berry KP, Subramanian J, Cha JW, Oh WC, Kwon HB, Kubota Y, So PT, Nedivi E (2016). Inhibitory synapses are repeatedly assembled and removed at persistent sites in vivo. Neuron.

[5] Xue Y, Berry KP, Boivin JR, Wadduwage D, Nedivi E, So PTC (2018). Scattering reduction by structured light illumination in line-scanning temporal focusing microscopy. Biomed. Opt. Express.

[6] Dana H, Marom A, Paluch S, Dvorkin R, Brosh I, Shoham S (2014). Hybrid multiphoton volumetric functional imaging of large-scale bioengineered neuronal networks. Nat. Commun..

[7] Zhu G, van Howe J, Durst M, Zipfel W, Xu C (2005). Simultaneous spatial and temporal focusing of femtosecond pulses. Opt. Express.

[8] Oron D, Tal E, Silberberg Y (2005). Scanningless depth-resolved microscopy. Opt. Express.

[9] Tal E, Oron D, Silberberg Y (2005). Improved depth resolution in video-rate line-scanning multiphoton microscopy using temporal focusing. Opt. Lett..

[10] Kim KH, Buehler C, Bahlmann K, Ragan T, Lee W-CA, Nedivi E, Heffer EL, Fantini S, So PTC (2007). Multifocal multiphoton microscopy based on multianode photomultiplier tubes. Opt. Express.

[11] Gigan S, Katz O, de Aguiar HB, Andresen ER, Aubry A, Bertolotti J, Bossy E, Bouchet D, Brake J, Brasselet S, Bromberg Y, Cao H, Chaigne T, Cheng Z, Choi W, Čižmár T, Cui M, Curtis VR, Defienne H, Hofer M, Horisaki R, Horstmeyer R, Ji N, LaViolette AK, Mertz J, Moser C, Mosk AP, Pégard NC, Piestun R, Popoff S, Phillips DB, Psaltis D, Rahmani B, Rigneault H, Rotter S, Tian L, Vellekoop IM, Waller L, Wang L, Weber T, Xiao S, Xu C, Yamilov A, Yang C, Yılmaz H (2022). Roadmap on wavefront shaping and deep imaging in complex media. J. Phys. Photonics.

[12] Cui M, McDowell EJ, Yang C (2010). An in vivo study of turbidity suppression by optical phase conjugation (TSOPC) on rabbit ear. Opt. Express.

[13] Jang M, Ruan H, Vellekoop IM, Judkewitz B, Chung E, Yang C (2015). Relation between speckle decorrelation and optical phase conjugation (OPC)-based turbidity suppression through dynamic scattering media: a study on in vivo mouse skin. Biomed. Opt. Express.

[14] Ruan H, Jang M, Judkewitz B, Yang C (2014). Iterative time-reversed ultrasonically encoded light focusing in backscattering mode. Sci. Rep..

[15] Kang S, Jeong S, Choi W, Ko H, Yang TD, Joo JH, Lee J-S, Lim Y-S, Park Q-H, Choi W (2015). Imaging deep within a scattering medium using collective accumulation of single-scattered waves. Nat. Photonics.

[16] Qureshi MM, Brake J, Jeon H-J, Ruan H, Liu Y, Safi AM, Eom TJ, Yang C, Chung E (2017). In vivo study of optical speckle decorrelation time across depths in the mouse brain. Biomed. Opt. Express..

[17] Choi H, Yew EYS, Hallacoglu B, Fantini S, Sheppard CJR, So PTC (2013). Improvement of axial resolution and contrast in temporally focused widefield two-photon microscopy with structured light illumination. Biomed. Opt. Express.

[18] Li Z, Hou J, Suo J, Qiao C, Kong L, Dai Q (2017). Contrast and resolution enhanced optical sectioning in scattering tissue using line-scanning two-photon structured illumination microscopy. Opt. Express.

[19] Wei Z, Boivin JR, Xue Y, Burnell K, Wijethilake N, Chen X, So PTC, Nedivi E, Wadduwage DN (2023). De-scattering deep neural network enables fast imaging of spines through scattering media by temporal focusing microscopy. Res. Sq..

[20] Escobet-Montalbán A, Spesyvtsev R, Chen M, Saber WA, Andrews M, Simon Herrington C, Mazilu M, Dholakia K (2018). Wide-field multiphoton imaging through scattering media without correction. Sci. Adv..

[21] Zheng C, Park JK, Yildirim M, Boivin JR, Xue Y, Sur M, So PTC, Wadduwage DN (2021). De-scattering with Excitation Patterning enables rapid wide-field imaging through scattering media. Sci Adv..

[22] Wijethilake N, Anandakumar M, Zheng C, So PTC, Yildirim M, Wadduwage DN (2023). DEEP-squared: Deep learning powered de-scattering with excitation patterning. Light Sci. Appl..

[23] Holtmaat A, Svoboda K (2009). Experience-dependent structural synaptic plasticity in the mammalian brain. Nat. Rev. Neurosci..

[24] van Versendaal D, Rajendran R, Saiepour MH, Klooster J, Smit-Rigter L, Sommeijer J-P, De Zeeuw CI, Hofer SB, Heimel JA, Levelt CN (2012). Elimination of inhibitory synapses is a major component of adult ocular dominance plasticity. Neuron.

[25] Denk W, Strickler JH, Webb WW (1990). Two-photon laser scanning fluorescence microscopy. Science.

[26] Helmchen F, Denk W (2005). Deep tissue two-photon microscopy. Nat. Methods.

[27] Azimipour M, Atry F, Pashaie R (2015). Effect of blood vessels on light distribution in optogenetic stimulation of cortex. Opt. Lett..

[28] Yona G, Meitav N, Kahn I, Shoham S (2016). Realistic numerical and analytical modeling of light scattering in brain tissue for optogenetic applications(1,2,3). eNeuro.

[29] Min E, Ban S, Wang Y, Bae SC, Popescu G, Best-Popescu C, Jung W (2017). Measurement of multispectral scattering properties in mouse brain tissue. Biomed. Opt. Express.

[30] Dunn AK, Wallace VP, Coleno M, Berns MW, Tromberg BJ (2000). Influence of optical properties on two-photon fluorescence imaging in turbid samples. Appl. Opt..

[31] Subramanian J, Dye L, Morozov A (2013). Rap1 signaling prevents L-type calcium channel-dependent neurotransmitter release. J. Neurosci..

[32] Lee WC, Chen JL, Huang H, Leslie JH, Amitai Y, So PT, Nedivi E (2008). A dynamic zone defines interneuron remodeling in the adult neocortex. Proc. Natl. Acad. Sci. USA.

