# Supplementary material for: Multiline orthogonal scanning temporal focusing (mosTF) microscopy for scattering reduction in in vivo brain imaging
Source: Sci Rep. 2024 May 13;14:10954. doi: 10.1038/s41598-024-57208-6 (PMC11091065; doi:10.1038/s41598-024-57208-6)
Supplement: Supplementary file 1 — Supplementary Figures. [file 41598_2024_57208_MOESM1_ESM.pdf]

Multiline Orthogonal Scanning Temporal Focusing (mosTF) Microscopy for Scattering Reduction in High-speed *in vivo* Brain Imaging

Yi Xue[1,2,8], Josiah R. Boivin[3], Dushan N. Wadduwage[2,4,7], Jong Kang Park[4], Elly Nedivi[3,5,6] and Peter T.C. So[1,2,4,*]

[1]Dept. of Mechanical Engineering, [2]Laser Biomedical Research Center, [3]Picower Institute, [4]Dept. of Biological Engineering, [5]Dept. of Biology, [6]Dept. of Brain and Cognitive Sciences, Massachusetts Institute of Technology 77 Massachusetts Ave., Cambridge MA 02139. [7]Center for Advanced Imaging, Faculty of Arts and Sciences, Harvard University, Cambridge, MA 02138. [8]Dept. of Biomedical Engineering, University of California, Davis, CA 95616, USA.

**ptso@mit.edu*

Supplementary materials

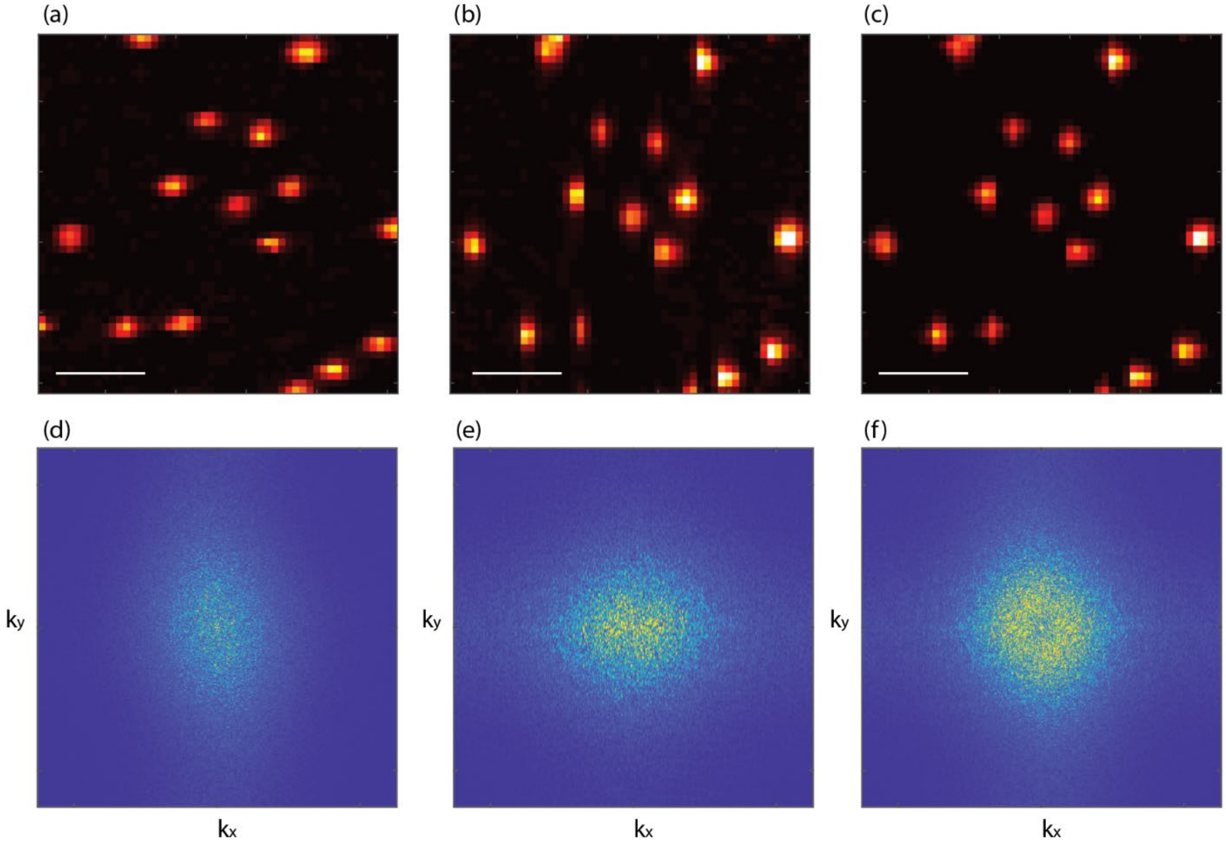

Figure S1. Orthogonal scanning provides an isotropic PSF. (a) Reconstructed image of 200 nm beads from horizontal scanning only. (b) Reconstructed image of 200 nm beads from vertical scanning only. (c) Combination of (a) and (b) to generate isotropic PSF. Scale bar of (a-c), 5 μm. Figures (a-c) are normalized to the same color scale. (d) Fourier transform of (a). The frequency coverage after reconstruction in the horizontal direction is extended in the vertical direction. (e) Fourier transform of (b). (f) Combination of (d) and (e) with a weighting factor to balance the frequency coverage in two directions. The frequency coverage is isotropic in the Fourier domain. The inverse Fourier transform of (f) generates (c).

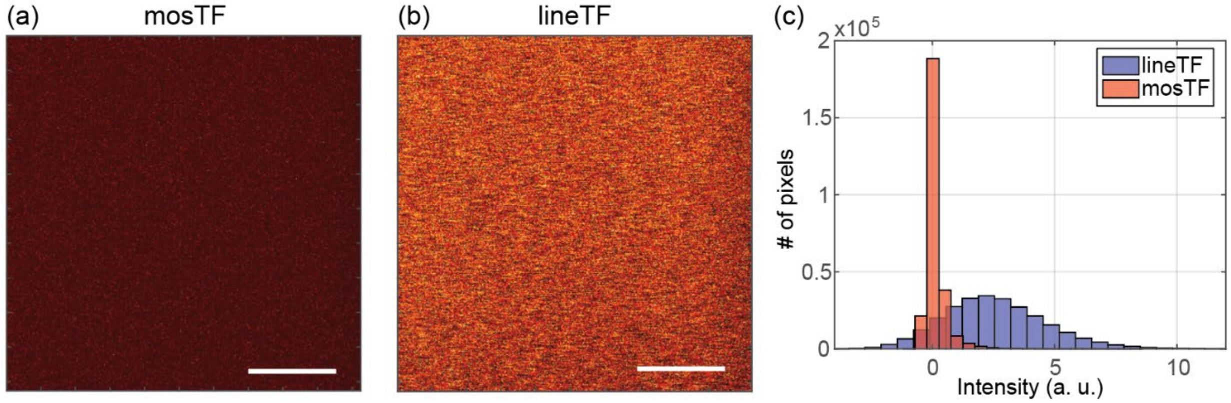

Figure S2. Background measurement of (a) mosTF and (b) lineTF. These two figures are normalized to the same color scale [-1 9]. Scale bar, 50 μm. (c) Pixel value histogram of mosTF and lineTF. The camera pixel has an offset of 300 counts to avoid negative value of intensity, which was subtracted during our calculation. The mean value of mosTF background is $0.13 \pm 0.39e^-$, and the mean value of lineTF background is $2.57 \pm 2.14e^-$.
